# Monitoring Nutrient Status of Brown Marmorated Stink Bug Adults and Nymphs on Summer Holly

**DOI:** 10.3390/insects9030120

**Published:** 2018-09-17

**Authors:** Victoria P. Skillman, Nik G. Wiman, Jana C. Lee

**Affiliations:** 1 OSU Extension Plant Pathology Laboratory, Hermiston Agricultural Research & Extension Center, 2121 S. 1st Street, Hermiston, OR 97838, USA; 2 OSU Department of Horticulture, 4109 Agriculture & Life Science Building, Corvallis, OR 97331, USA; nik.wiman@oregonstate.edu; 3 USDA ARS Horticultural Crop Research Unit, Corvallis, OR 97330, USA; jana.lee@ars.usda.gov

**Keywords:** *Halyomorpha halys*, nutritional ecology, nymphal development, lipid, glycogen, sugar

## Abstract

*Halyomorpha halys* (Stål), or brown marmorated stink bug (BMSB), has become a major pest and nuisance for both agricultural growers and homeowners since its arrival in North America and Europe. The nutritional ecology of BMSB is important for understanding its life history and rearing requirements. However, little is known about the nutritional status of wild populations, especially in the U.S. This research monitored the nutrient status of nymphal and adult BMSB collected from English holly in western Oregon. We measured their weight, nutrient index (weight/(prothorax × width)^3^), lipid, glycogen and sugar levels and egg load from May–September/October. First, glycogen and sugar levels of adults were often lowest sometime in June-August with a general increase by September. Meanwhile, their lipid levels varied without a discernible trend. Second, adult females had few eggs in May, with the highest egg load in June and July, and no eggs by September. Lastly, first and second nymphal instars were found in June, and fourth and fifth instars in September. Because nothing is known about the nutrient levels of nymphs, the reported values from this survey can assist future research on physiological responses of BMSB to treatments or environmental impacts in the field.

## 1. Introduction

The brown marmorated stink bug (BMSB), *Halyomorpha halys* (Stål), was first detected in the United States in 1996 and Europe in 2004 [1,2,3]. It has established in 44 U.S. states including Oregon in 2004 [4]. Native to eastern Asia, BMSB nymphs and adults feed on ~150 different plant species in the U.S. and damage the leaves, buds, stems and fruits [5]. In Oregon, BMSB are often found on English holly, maple, tree of heaven, empress tree, catalpa, ash, dogwood and Himalayan blackberries [6]. In temperate regions, BMSB have one generation per year, while in warmer tropical regions, up to five generations [2]. Adult BMSB overwinter in aggregations in man-made structures, making them a major nuisance to homeowners, as well [7].

Understanding the nutritional ecology of BMSB improves our knowledge of this pest’s life history. Prior work on the development of BMSB on different diets has enabled researchers to optimize rearing BMSB for research use [8,9,10,11]. Monitoring the energetic reserves of field-collected adults has revealed that extensive nutrient depletion occurs during overwintering. In spring, emerged BMSB adults have lower glycogen and sugar reserves than concurrently overwintering BMSB [12]. Furthermore, overwintering adults exhibit a 12–25% decline in lipid, a 48–70% decline in glycogen and a 54–79% decline in sugar levels from October–June [12]. Given that BMSB have low reserves upon emergence from diapause, adults must feed on host plants to replenish their reserves. Insects may use either lipid, glycogen, sugar or a combination of these reserves for diapause, reproduction or flight [13,14,15]. To date, there is little information on the nutrient reserves of adult BMSB once they access host plants during spring and summer. Therefore, the first objective of our study was to monitor the weight, nutrient index (weight/(prothorax × width)^3^) [16], lipid, glycogen and sugar reserves of wild BMSB adults from May–October on English holly trees, *Ilex aquifolium* L., over two years.

This first objective on nutrient profiles of BMSB was based on a preliminary study in 2012–2014 [17]. In that study, BMSB adults were collected from wild and ornamental host plants from March–October in western Oregon to measure their lipid, glycogen and sugar levels. Two trends were observed: female BMSB had higher values than males, and glycogen and sugar levels were lower in mid- to late summer. However, in that study, collection sites and host plants varied throughout the collection season. Adults from different host plants may have different nutrient levels; nymphal diet has affected the sugar, lipid and protein levels in eclosed adults in the laboratory [18]. Therefore, this new study examined seasonal variation by sampling regularly from five sites with high BMSB populations on one preferred host, English holly, where BMSB of all life stages are frequently found resting or feeding [6]. BMSB prefer to colonize hosts bearing fruit [19], and English holly has fruits present through the collection period.

Understanding the reproductive phenology of BMSB is essential to develop predictive models that assist growers to predict pest occurrence. While reproductive development on BMSB has been recorded in multiple states and crops [20], this information has not been examined in the context of their nutrient reserves. Thus, the second objective was to monitor the egg load of field-collected female BMSB and examine the relationship with nutrient status during summer.

Monitoring the activity of nymphal instars of BMSB is important for pest control. Insecticide applications against nymphs can be more efficacious because soft-bodied nymphs are more susceptible than adults [21]. Furthermore, several native insects prey on BMSB nymphs based on a study in several Mid-Atlantic States [22] and a study in Oregon [23]. Sand wasps, *Bicyrtis quadrifasciatus* Say (Hymenoptera: Crabronidae) and *Astata unicolor* Say (Hymenoptera: Crabronidae), prefer to raise their young on live nymphal BMSB. *Bicyrtis quadrifasciatus* nests contained on average over 50 BMSB nymphs. Currently, the nutritional value of BMSB nymphs have not been measured, whereas BMSB egg masses are known to provide ~25.5 μg of lipid, 3 µg glycogen and 3 µg of sugar per egg [24]. Therefore, our third objective was to monitor the seasonal occurrence of BMSB nymphal instars on English holly from spring to fall while measuring their nutrient reserves.

This monitoring paper highlights potential seasonal variation among the nutrient status of adult BMSB, describes the nutritional content of nymphal BMSB to predators and provides baseline information for future studies examining BMSB nutritional ecology in various habitats, management or environmental conditions.

## 2. Materials and Methods

### 2.1. Collection

BMSB adults and nymphs were collected from holly trees at five sites in the Willamette Valley of Oregon: Corvallis (44°31′49″ N 123°16′09″ W), Albany (44°37′53″ N 123°07′09″ W), Aurora (45°13′42″ N 122°47′09″ W), Molalla (45°11′42″ N 122°34′54″ W) and Monmouth (44°50′55″ N 123°14′20″ W). Sites had ornamental or commercial plantings of holly where BMSB were collected by beat sheet at least twice a month per site from May–September or October when adults were present. Nymphal instars were only collected in 2015. BMSB were transported live in well-ventilated mesh containers with a water wick from the field and quickly frozen at −80 °C in the lab.

### 2.2. Nutrient Bioassay

After removal from the −80 °C freezer, all BMSB were weighed, and the prothorax width was measured to calculate the nutrient index (body weight/(prothorax × width)^3^ (mg/mm^3^)), which is a body mass index for BMSB used previously to evaluate pre- and post-overwintering physiological status [16]. Adult female BMSB were dissected to count eggs. Reproductive status was also ranked pre- to post-vitellogenic according to Nielsen et al. (2017) [20] based on the presence of immature and mature eggs and the appearance of the spermatheca and ovaries. Next, all parts of the dissected female including eggs were transferred back into the tube for nutrient assays for this study. While separate nutrient assays can be run on the eggs and remaining body [24], this was not practical for the large numbers of bugs processed. These eggs must be carefully separated from tissue to avoid rupturing them.

The lipid, glycogen and total sugar content of individual BMSB were quantified using a protocol developed for mosquitoes [25,26]. The vanillin assay reacts with lipids such as triglycerides and fat droplets [26]. The anthrone assay reacts with sugars (i.e., fructose, glucose, sucrose, trehalose) and glycogen [25]. These procedures have been adapted for coccinellids [27], a drosophilid fly [28], a parasitic wasp [29], a phorid fly [30] and a tephritid fly [31]. Prior hydration will not affect nutrient readings because they are measured per individual, and not per weight of each individual. Because of BMSB’s large body size, some modifications to the protocol were necessary and are described here. Each individual BMSB was crushed with a pestle in a 1.5-mL microcentrifuge tube and 110 µL of 2% sodium sulfate. A control that included all reagents, but no BMSB, was run during each assay set of 20 BMSB. Next, 990 µL of chloroform-methanol (1:2) were added, and the tube was centrifuged for 3 min at 16,000× *g* to collect the glycogen precipitate. For adults, the supernatant (~1000 µL) was transferred into a glass test tube, vortexed and aliquoted further: 50 µL for the lipid assay and 50 µL for the sugar assay. For nymphs, the final aliquots of the supernatant used to react for sugar and lipid assays was based on nymphal instar (Table 1). The multiplier used to estimate the resulting nutrient content on the whole body of a nymph was adjusted accordingly for each nymphal instar (Table 2).

For the lipid assay, the supernatant was boiled at 90 °C for ~2 min until the liquid evaporated. Next, 40 µL of sulfuric acid were added, and the mixture was heated at 90 °C for 2 min. Once cooled, 960 µL of vanillin reagent were added, vortexed and left at room temperature for 20–30 min. The solution was poured into a cuvette, and absorbance was read at 525 nm on a spectrophotometer (Ultrospec 3100 pro, Amersham Biosciences, Piscataway, NJ, USA). Lipid content was estimated from the absorbance values of lipid standards made for each vanillin reagent. To calibrate the standard, 0, 1, 5, 10, 35 and 50 µg of canola oil were reacted with vanillin as described above, and the relationship between the absorbance value and lipid content was calculated by a linear equation. Similar calibrations were done for glycogen and sugar standards. For the glycogen assay, 975 µL of anthrone reagent were added and vortexed until the precipitate dissolved. For adults, an aliquot of 50 µL of this mixture was transferred to a new tube, to which an additional 950 µL anthrone was added. For nymphs, the aliquot of supernatant was taken based on nymphal instar (Table 1), and a corresponding amount of anthrone was added to reach a final volume of 1000 µL (Table 2). The solution was vortexed, then heated at 90 °C for 10 min. Once cooled, absorbance was read at 625 nm to determine glycogen levels. For the sugar assay, each tube was heated at 90 °C for 1 min, leaving ~25 µL of supernatant. Next, 975 µL of anthrone reagent was added, and then, tubes were vortexed and heated at 90 °C for 10 min. Once the solution cooled, absorbance was read at 625 nm to determine sugar levels. Because a certain aliquot of the 1000 µL supernatant or first glycogen mixture was used to assess the lipid, glycogen and sugar level, the estimate was multiplied accordingly (Table 2) for calculating total levels in individual BMSB.

### 2.3. Statistical Analyses

To simplify the models for adult BMSB, each sex and year were run independently. Females were expected to have higher nutrient levels and weight than males [17]. The weight, nutrient index, lipid, glycogen, sugar content and nutrient content/weight of each stink bug were compared separately as response variables. A lognormal distribution was used in a generalized linear mixed model in PROC GLIMMIX [32]. Month was a fixed effect, while the site where BMSB adults were collected was a random effect. Prothorax width was a covariate in all analyses with lipid, glycogen or sugar as the response variable, but not with weight or nutrient index. Post-hoc comparisons of months were done by Tukey HSD.

The relationships between the egg load and nutrient reserves were tested by regression in PROC REG [28]. A regression was run for each combination of two variables (egg load, weight, nutrient index, lipid, glycogen and sugar) for females and males collected in each year.

No statistics were run for the nutrient levels of nymphal instars because this information was intended to provide baseline measurements for the five instars. Furthermore, different nymphal instars were collected each month, so observed differences between instars could also be influenced by varying conditions at the time of collection.

## 3. Results

### 3.1. Adult Nutrient Profiles

**Figure 1:**
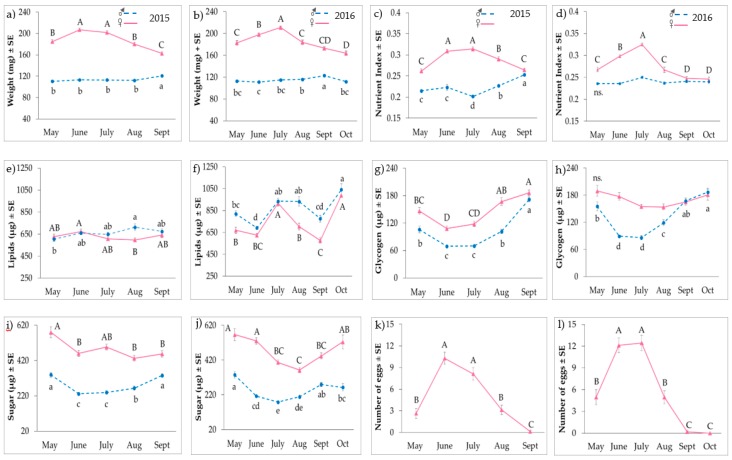
Average (±SE) weight (**a**,**b**); nutrient index (**c**,**d**); lipid (**e**,**f**); glycogen (**g**,**h**); sugar (**i**,**j**) and egg count (**k**,**l**) of BMSB adults collected from holly in Oregon in 2015–2016, respectively. Upper and lower case letters indicate monthly differences by Tukey HSD among females and males, respectively. Nutrient levels per wet weight in Table S1.

Results are presented per individual (Figure 1) and per mg (Table S1) to facilitate observations across sex, but these latter values may be influenced by insect hydration. There were significant differences between months for weight, nutrient index and the three nutrients over the summer for both sexes in 2015 and 2016 (Table 3). The weight of males remained fairly constant at 111–123 mg throughout the season, while females experienced an increase in body weight in June or July (Figure 1a,b). This same increase was observed over this period in the nutrient index for males and females (Figure 1c,d).

Glycogen and sugar levels dipped in June–August in seven out of eight trend lines (Figure 1g–j). Generally, carbohydrate levels increased again in September and October, except for female sugar in 2015 and female glycogen in 2016. The lipid levels did not show a consistent trend and varied the most between years (Figure 1e,f). Lipid levels differed by 77 and 108 µg between months among females and males, respectively (Figure 1e), but differed by 423 and 350 µg between months in 2016 (Figure 1f). When nutrients were examined on a per mg basis (Table S1), glycogen and sugar levels were ~1 µg/mg and 2–3 µg/mg, respectively. Lipids were ~3 µg/mg among females and 5–9 µg/mg among males.

Lipid levels did not significantly vary with glycogen or sugar levels, or only weakly with low *r*^2^-values of <0.05 (Table 4). Glycogen levels significantly increased as sugar levels in an individual also increased, with moderate *r*^2^-values of 0.227–0.478.

### 3.2. Reproductive Status

The egg load for the females peaked in June and July (Figure 1k,l). The ovarial and spermathecal ranks followed the egg load trend, with females having non-productive ranks in May, September and October and higher reproduction ranks in June and July (Tables S1 and S2). The number of individuals collected both years by location and by sex is reported in Table S3.

Female egg load significantly correlated with individual weight and nutrient index (Table 4), with modest *r*^2^-values of 0.25–0.34. Egg load of females had no or minimal relationships with total lipid, glycogen or sugar levels measured from the entire female, which included eggs. The regressions were not significant or had very low *r*^2^-values of <0.01. There was a weak negative association in 2015; as egg load increased, total lipid or glycogen levels decreased.

### 3.3. Nymphal BMSB

**Figure 2:**
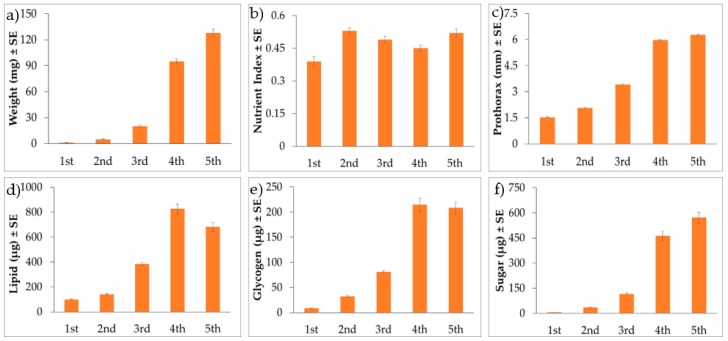
Average (± SE) of weight (**a**); nutrient index (**b**); prothorax (**c**); lipid (**d**); glycogen (**e**) and sugar (**f**) of the five nymphal instars of BMSB collected in 2015. Nutrient levels per wet weight in Table S1.

A total of 605 nymphs were collected over the 2015 summer. The percentage of nymphs per month was tracked over the summer months of June–September (Table 5). In June, only first and second instars were collected; while in September, fourth and fifth nymphal instars predominated. In the mid-summer of July and August, a mix of nymphal instars was found. Average nutrient content was observed to increase with instar stage (Figure 2).

As expected, there was an increase in total lipids, glycogen and sugar level, weight and prothorax width with the progression of nymphal instars (Figure 2). Not surprisingly, first instars were observed with lower nutrient levels, while fifth instars often with the highest. The nutrient index fluctuated some between the fourth and fifth nymphal instars. Like adults, lipids were most abundant in nymphs, followed by sugar and then glycogen. On a per body mass basis, lipid levels were ~77 µg/mg among first instars to 6 µg/mg among fifth instars (Table S1). Glycogen levels were ~2–7 µg/mg, and sugar levels were ~4–8 µg/mg.

## 4. Discussion

### 4.1. Adult BMSB

The nutrient levels of BMSB adults collected from holly fluctuated from May–September/October. Generally, sugar and glycogen levels dipped around July. Notably, the dip occurred with repeated sampling from five sites and one host type over two summers and is consistent with observations from more random sampling across 12 sites in Northwestern Oregon from multiple hosts in 2012–2014 [17]. First, an explanation for these fluctuations is that it reflects the generational change in the population dynamics. As the overwintered generation of adults expend their energy in summer, they later die off in July/August upon which the newly emerging adults are often sampled with higher energetic reserves [12]. Secondly, another explanation for the fluctuations in nutrient levels could be the quality of food available and adult movement at different times of the summer. BMSB are very mobile creatures [33,34,35], so the adults collected from holly may have fed solely on holly or may have migrated from other hosts. Fluctuations in nutrient levels may reflect migrations of BMSB depleted of nutrient reserves or having fed on hosts of lower quality. Thirdly, other environmental factors could influence BMSB metabolism and development, such as temperature or precipitation [36]. The dip in carbohydrate levels occurred during the heat of the summer when their metabolic rates might be higher. Lastly, the carbohydrate dip could have resulted from several factors interacting with each other.

Lipid levels showed substantial variation, which may reflect the numerous metabolic pathways associated with lipids [14]. Females had 3 µg of lipid per mg of body mass, and males had 5–9 µg/mg, whereas both sexes had overlapping carbohydrate levels per mg. This discrepancy should be investigated further, particularly if egg production demands among females might lower the amount of available lipids [37]. Furthermore, lipid content of adults did not or was only weakly associated with glycogen and sugar reserves. Meanwhile, glycogen and sugar levels positively associated with each other. This may be expected since glucose can be converted to glycogen for storage, and glycogen can be exported as trehalose, a common hemolymph sugar [38]. While it was not the scope of this survey, additional studies on BMSB nutritional ecology should examine trends in the context of changing environmental factors and nutritional quality of hosts [8,12].

### 4.2. Reproductive Status

The egg load, ovarial and spermathecal ranks of female BMSB collected from holly consistently showed that females were beginning reproduction in May, peaked in June–July and were in reproductive diapause by September in 2015–2016. This trend is consistent with previous BMSB collections from the Willamette Valley of Oregon, from 2012–2014 from a range of hosts [20].

Females with greater egg loads often weighed more, or had higher nutrient index values, which may reflect the added weight of eggs. The relationship between female egg load and total lipid, glycogen or sugar content was not as clear. Females with more eggs tended to have lower lipid levels, although this trend was weak. In another pentatomid species, ovarian development was observed to coincide with decreased body fat [37]. It is possible that such a trend may become clearer if dissected BMSB females had their eggs carefully picked out, and nutrient assays were run on their remaining body parts (and not the entire body contents). Since BMSB eggs are primarily comprised of lipids [24], females that invested in egg production may have lower fat body reserves.

### 4.3. Nymphal BMSB

The progression of nymphal instars from June–September 2015 suggests that there was just one generation per year on holly hosts. Previous trends observed on *Paulownia tomentosa* also suggested a univoltine population in the Northeastern U.S. [39]. Nymphs were present starting in June, which corresponds with a large number of the adults leaving their overwintering sites in mid-May and laying eggs. The presence of large nymphs in the summer corresponds with the fact that much of the F_1_ generation on holly may not be reproductively mature before fall weather sets in and they start moving to overwintering sites.

As expected, lipid, glycogen and sugar levels progressively rose with the nymphal instars. Average nutrient levels among the fourth and fifth nymphal instars sometimes overlapped. Smaller instars were observed to have high lipid content per mg of body mass compared to larger instars. This suggests that smaller BMSB have a greater density of lipids in their body.

## 5. Conclusions

BMSB is an important pest across North America and Europe, and exploring the nutrient status of wild populations provides insight on its life history and nutritional quality as prey. Adult BMSB nutrient levels fluctuate through spring to fall with glycogen and sugar levels often dipping in summer. BMSB may be univoltine on holly; first and second instars were present in June, and fourth and fifth instars in September. Among adults and all nymphal instars, lipids were the predominant energy reserve, followed by sugar, and glycogen the lowest. Future studies should examine how different hosts or management tactics affect the energetic reserves of BMSB in the landscape.

## Figures and Tables

**Table 1:** Aliquot taken from the supernatant for lipid and sugar reading and aliquot taken from the first glycogen-anthrone mixture for glycogen readings based on the nymphal instar of brown marmorated stink bug (BMSB) nymph.

Nymphal	Aliquot of Supernatant or Glycogen Mixture		
Instar	Lipid	Glycogen	Sugar
5th	50 µL	50/100 µL	50 µL
4th	50 µL	100 µL	50 µL
3rd	50/100 µL	100/150 µL	50/100 µL
2nd	150/250 µL	200/400 µL	150/250 µL
1st	300 µL	500 µL	300 µL

**Table 2:** The evaporation time for lipid and sugar reading, amount of additional anthrone added for glycogen reading and multiplier based on amount aliquoted for a BMSB nymph.

Amount Aliquoted	Lipid and Sugar Evaporation Time	Additional Anthrone Added to Glycogen Aliquot	Multiplier Based on Amount Aliquoted
50 µL	1 min	950 µL	20
100 µL	2 min	900 µL	10
150 µL	3 min	850 µL	6.667
200 µL	4 min	800 µL	5
250 µL	5 min	750 µL	4
300 µL	6 min	700 µL	3.333
400 µL	7 min	600 µL	2.5
500 µL	8 min	500 µL	2

**Table 3:** The effect of month on weight, nutrient index, lipid, glycogen, sugar and egg of summer female and male adult BMSB in Oregon in 2015–2016, with prothorax as a covariate.

Year	Measurement	Effects	Female			Male		
			*df*	*F*	*p*	*df*	*F*	*p*
2015	Weight	Month	4633	41.60	<0.0001	4661	9.32	<0.0001
	Nutrient Index	Month	4633	30.83	<0.0001	4661	71.82	<0.0001
	Lipid	Month	4612	2.06	0.0844	4660	2.49	0.0419
		Prothorax	1612	0.59	0.4432	1660	2.53	0.1124
	Glycogen	Month	4632	25.89	<0.0001	4660	94.38	<0.0001
		Prothorax	1632	0.04	0.8452	1660	6.62	0.0103
	Sugar	Month	4632	4.50	0.0014	4660	38.59	<0.0001
		Prothorax	1632	7.98	0.0049	1660	47.22	<0.0001
	Egg	Month	4632	280.85	<0.0001	.	.	.
2016	Weight	Month	5541	28.37	<0.0001	5562	9.89	<0.0001
	Nutrient Index	Month	5541	53.59	<0.0001	5562	0.88	0.4939
	Lipid	Month	5540	30.56	<0.0001	5561	13.25	<0.0001
		Prothorax	1540	4.26	0.0396	1561	1.38	0.2411
	Glycogen	Month	5540	1.89	0.0943	5561	52.30	<0.0001
		Prothorax	1540	3.19	0.0748	1561	10.50	0.0013
	Sugar	Month	5540	1.89	0.0943	5561	24.28	<0.0001
		Prothorax	1540	17.89	<0.0001	1561	11.07	0.0009
	Egg	Month	5540	39.41	<0.0001	.	.	.

**Table 4:** Regression analyses of BMSB egg load and nutrient values.

Year-Sex	Variable (*x*)	Variable (*y*)	*df*	*F*	*p*	*r* ^2^	Slope	*y*-Intercept
2015-females	Egg load	Weight	1620	278.30	<0.0001	0.310	0.00242	0.175
	Egg load	Nutrient Index	1620	201.10	<0.0001	0.245	0.00314	0.274
	Egg load	Lipid	1620	7.73	0.0056	0.012	−2.984	645.2
	Egg load	Glycogen	1620	4.43	0.0358	0.007	−0.7696	148.6
	Egg load	Sugar	1620	1.32	0.251	.	.	.
2015-males	Glycogen	Lipid	1620	15.10	<0.0001	0.024	−0.452	696.2
	Sugar	Lipid	1620	30.90	<0.0001	0.048	−0.220	735.5
	Sugar	Glycogen	1620	211.00	<0.0001	0.254	0.173	62.43
	Glycogen	Lipid	1668	4.07	0.0442	0.006	−0.305	696.4
	Sugar	Lipid	1668	20.70	<0.0001	0.030	−0.365	766.4
	Sugar	Glycogen	1668	392.00	<0.0001	0.370	0.327	15.27
2016-females	Egg load	Weight	1549	219.50	<0.0001	0.286	0.0018	0.176
	Egg load	Nutrient Index	1549	277.10	<0.0001	0.336	0.0030	0.259
	Egg load	Lipid	1549	2.48	0.116	.	.	.
	Egg load	Glycogen	1549	1.47	0.226	.	.	.
	Egg load	Sugar	1549	5.55	0.0188	0.01	2.20	452.65
2016-males	Glycogen	Lipid	1549	0.78	0.38	.	.	.
	Sugar	Lipid	1549	21.20	<0.0001	0.037	−0.264	848.0
	Sugar	Glycogen	1549	161.00	<0.0001	0.227	0.178	85.93
	Glycogen	Lipid	1571	0.01	0.959	.	.	.
	Sugar	Lipid	1571	27.20	<0.0001	0.046	−0.573	985.3
	Sugar	Glycogen	1571	522.00	<0.0001	0.478	0.407	28.61

**Table 5:** The percentage and number of individuals per nymphal instar collected each month in 2015.

Nymphal Instar	June		July		August		September	
**1st**	61.9%	52	1.4%	4	.	.	.	.
**2nd**	38.1%	32	43.4%	124	6.9%	12	.	.
**3rd**	.	.	50.7%	145	40.2%	70	.	.
**4th**	.	.	.	.	31.6%	55	66.7%	30
**5th**	.	.	4.5%	13	21.3%	37	33.3%	15
**Total**	.	84	.	286	.	174	.	45

## Supplementary Materials

The following are available online at http://www.mdpi.com/2075-4450/9/3/120/s1, Table S1: The average nutrient level per mg (±SE) for adult, female and male, and nymphal BMSB. Different letters denote a significant difference between months by Tukey HSD for each nutrient-sex-year grouping (statistical outputs similar to Table 3, not shown); no statistics on nymphs. Table S2: The number (*n*) and percent (%) of females per ovarial rank (1 undeveloped, 2 reproductive mature, but not mated, 3 mated with starting of egg development, 4 high egg count with filled mature eggs, 5 post egg laid) by month and year for summer-collected BMSB. Table S3: The number (*n*) and percent (%) of female per spermathecal rank (1 unmated, 2 unsure, 3 mated) by month and year for the summer-collected BMSB, Table S4: The number of females and males collected at each of the five locations for the 2015–2016 summer collection of BMSB adults.
