# Supplementary material for: Monitoring Nutrient Status of Brown Marmorated Stink Bug Adults and Nymphs on Summer Holly
Source: Insects. 2018 Sep 17;9(3):120. doi: 10.3390/insects9030120 (PMC6164943; doi:10.3390/insects9030120)
Supplement: Supplementary file 1 [file insects-09-00120-s001.pdf]

Supplementary Materials for "Monitoring Nutrient Status of Brown Marmorated Stink Bug Adults and Nymphs on Summer Holly" by Skillman, Wiman and Lee in "Insects"

Table S1. The average nutrient level per mg (± SE) for adult, female and male, and nymphal BMSB. Different letters denote significant difference between months by Tukey HSD for each nutrient-sex-year grouping (statistical outputs similar to Table 3, not shown); no statistics on nymphs.

**Collection group**	**Female** Lipid (μg/mg)	Glycogen	Sugar	**Male** Lipid	Glycogen	Sugar
May 2015	3.5 ± 0.1 ab	0.82 ± 0.05 b	3.2 ± 0.18 a	5.4 ± 0.2 ab	1.0 ± 0.08 b	3.1 ± 0.13 a
June	3.3 ± 0.1 abc	0.53 ± 0.02 c	2.2 ± 0.08 c	5.9 ± 0.2 a	0.62 ± 0.03 c	2.1 ± 0.07 b
July	3.1 ± 0.1 c	0.59 ± 0.03 c	2.5 ± 0.07 bc	5.8 ± 0.2 a	0.62 ± 0.03 c	2.1 ± 0.07 b
August	3.5 ± 0.1 bc	0.97 ± 0.05 b	2.5 ± 0.12 bc	6.3 ± 0.2 a	0.90 ± 0.04 b	2.3 ± 0.1 b
September	3.6 ± 0.2 a	1.1 ± 0.04 a	2.9 ± 0.12 b	5.7 ± 0.2 b	1.4 ± 0.04 a	2.8 ± 0.09 a
May 2016	3.4 ± 0.1 bcd	0.82 ± 0.05 a	3.2 ± 0.18 a	7.4 ± 0.3 b	1.4 ± 0.08 b	2.9 ± 0.16 a
June	3.3 ± 0.1 d	0.52 ± 0.02 ab	2.2 ± 0.08 a	6.3 ± 0.2 c	0.81 ± 0.03 d	1.9 ± 0.05 bc
July	3.1 ± 0.09 b	0.59 ± 0.28 b	2.5 ± 0.07 b	8.2 ± 0.3 ab	0.75 ± 0.04 d	1.5 ± 0.05 d
August	3.5 ± 0.1 bc	0.97 ± 0.05 ab	2.5 ± 0.12 b	8.0 ± 0.4 ab	1.0 ± 0.05 c	1.8 ± 0.09 cd
September	3.6 ± 0.2 cd	1.2 ± 0.05 a	2.9 ± 0.13 a	6.5 ± 0.3 c	1.4 ± 0.05 b	2.3 ± 0.09 b
October	3.4 ± 0.4 a	1.2 ± 0.09 a	1.9 ± 0.2 a	9.4 ± 0.5 a	1.7 ± 0.07 a	2.3 ± 0.17 b
**Instar**	**Nymphs**					
1st	76.5 ± 5.3	6.4 ± 0.4	4.1 ± 0.37			
2nd	30.2 ± 1.4	6.8 ± 0.4	7.8 ± 0.4			
3rd	23.4 ± 0.9	4.8 ± 0.2	6.1 ± 0.2			
4th	9.8 ± 0.6	2.3 ± 0.1	4.6 ± 0.2			
5th	6.1 ± 0.5	1.6 ± 0.1	4.4 ± 0.2			

Table S2. The number (*n*) and percent (%) of females per ovarial rank (1 undeveloped, 2 reproductive mature, but not mated, 3 mated with starting of egg development, 4 high egg count with filled mature eggs, 5 post egg laid) by month and year for summer-collected BMSB.

Year	Ovarial Rank	May		June		July		August		September		October	
		n	%	*n*	%	*n*	%	*n*	%	*n*	%	*n*	%
2015	1	74	74.7	17	12.4	10	8.7	81	62.3	143	88.8	0	0.0
	2	10	10.1	28	20.4	12	10.4	19	14.6	16	9.9	0	0.0
	3	6	6.1	47	34.3	33	28.7	12	9.2	0	0.0	0	0.0
	4	9	9.1	41	29.9	26	22.6	11	8.5	1	0.6	0	0.0
	5	0	0.0	4	2.9	34	29.6	7	5.4	1	0.6	0	0.0

	Total	99	.	137	.	115	.	130	.	161	.	0	.
2016	1	35	43.8	4	3.6	10	8.7	50	56.8	111	96.5	52	100.0
	2	26	32.5	25	22.7	12	10.4	0	0.0	1	0.9	0	0.0
	3	6	7.5	31	28.2	33	28.7	17	19.3	1	0.9	0	0.0
	4	13	16.3	42	38.2	26	22.6	11	12.5	1	0.9	0	0.0
	5	0	0.0	8	7.3	34	29.6	10	11.4	1	0.9	0	0.0
	Total	80	.	110	.	115	.	88	.	115	.	52	.

Table S3. The number (*n*) and percent (%) of female per spermathecal rank (1 unmated, 2 unsure, 3 mated) by month and year for the summer collected BMSB.

Year	Sperm. Rank	May		June		July		August		September		October	
		n	%	*n*	%	*n*	%	*n*	%	*n*	%	*n*	%
2015	1	72	72.7	26	19.0	11	9.6	85	65.4	160	99.4	0	0.0
	2	8	8.1	20	14.6	12	10.4	14	10.8	0	0.0	0	0.0
	3	19	19.2	91	66.4	92	80.0	31	23.8	1	0.6	0	0.0
	Total	99	.	137	.	115	.	130	.	161	.	0	.
2016	1	58	72.5	15	13.6	0	0.0	49	55.7	111	96.5	52	100.0
	2	4	5.0	9	8.2	6	5.7	0	0.0	1	0.9	0	0.0
	3	18	22.5	86	78.2	100	94.3	39	44.3	3	2.6	0	0.0
	Total	80	.	110	.	106	.	88	.	115	.	52	.

Table S4. The number of adult female and male BMSB collected at each of the five locations from English holly during 2015–2016.

		May		June		July		August		September		October	
Sex	Location	2015	2016	2015	2016	2015	2016	2015	2016	2015	2016	2015	2016
Female	Albany	25	5	16	20	17	21	26	10	10	26	0	11
	Aurora	19	24	34	24	37	24	37	24	47	24	0	12
	Corvallis	11	10	19	23	11	20	7	12	40	23	0	11
	Molalla	19	33	33	24	32	24	34	24	32	24	0	12
	Monmouth	25	8	35	19	18	17	26	18	32	18	0	6
	Total	99	80	137	110	115	106	130	88	161	115	0	52
Males	Albany	15	9	20	21	14	22	24	16	25	22	0	5
	Aurora	20	25	34	24	35	24	37	24	46	24	0	12
	Corvallis	20	16	20	23	22	22	13	13	34	24	0	11
	Molalla	20	31	33	24	34	24	35	24	32	24	0	13
	Monmouth	25	10	35	23	22	23	23	22	32	12	0	6
	Total	100	91	142	115	127	115	132	99	169	106	0	47
